# Non-Muscle Myosin II in Axonal Cell Biology: From the Growth Cone to the Axon Initial Segment

**DOI:** 10.3390/cells9091961

**Published:** 2020-08-26

**Authors:** Ana Rita Costa, Monica M. Sousa

**Affiliations:** Nerve Regeneration Group, i3S-Instituto de Investigação e Inovação em Saúde and IBMC-Instituto de Biologia Molecular e Celular, Universidade do Porto, 4200-135 Porto, Portugal; arcosta@i3s.up.pt

**Keywords:** actin ring, actomyosin cytoskeleton, axon initial segment, axon growth, axon regeneration, growth cone, non-muscle myosin II

## Abstract

By binding to actin filaments, non-muscle myosin II (NMII) generates actomyosin networks that hold unique contractile properties. Their dynamic nature is essential for neuronal biology including the establishment of polarity, growth cone formation and motility, axon growth during development (and axon regeneration in the adult), radial and longitudinal axonal tension, and synapse formation and function. In this review, we discuss the current knowledge on the spatial distribution and function of the actomyosin cytoskeleton in different axonal compartments. We highlight some of the apparent contradictions and open questions in the field, including the role of NMII in the regulation of axon growth and regeneration, the possibility that NMII structural arrangement along the axon shaft may control both radial and longitudinal contractility, and the mechanism and functional purpose underlying NMII enrichment in the axon initial segment. With the advances in live cell imaging and super resolution microscopy, it is expected that in the near future the spatial distribution of NMII in the axon, and the mechanisms by which it participates in axonal biology will be further untangled.

## 1. Introduction

Myosins are one of the largest and most divergent protein families comprising over 35 classes (reviewed in [1,2]). In humans, myosins are encoded by 40 genes belonging to 13 different classes (I, II, III, V, VI, VII, IX, X, XV, XVI, XVIII, XIX, and XXXV) [1,2]. In reports from the early 1970s, in addition to striated and smooth muscle cells, myosins were shown to be present in several other cell types, including platelets, granulocytes, fibroblasts, and neurons (reviewed in [3]). Initially, these myosins were designated as vertebrate cytoplasmic myosins and nowadays are commonly referred to as non-muscle myosins (NM). Non-muscle myosin II (NMII) is present in every cell type and is the most abundant myosin class. The role of NMII in contraction has been broadly studied and is a crucial player in several key biological processes including cell migration, adhesion and cytokinesis, among many others. Actomyosin is the term used to refer to the cytoskeleton arrangement formed by the complex of actin and myosin. When NMII bipolar filaments bind anti-parallel actin filaments (F-actin), actomyosin contractility occurs through the conversion of chemical energy (hydrolysis of ATP) into mechanical energy, inducing myosin heads to move towards F-actin barbed (+) ends.

The actomyosin cytoskeleton is essential in neurons, where it is involved in the establishment of polarity, growth cone motility, axon growth and axon regeneration, radial and longitudinal axonal tension, and in the establishment of synapses. The details on how myosins, specifically NMII, participate in some of these processes are just starting to be unveiled as their comprehensive analysis is dependent on recent advances of microscopy. Here we will discuss the current knowledge on the participation of the actomyosin cytoskeleton on axonal biology and function. We will mainly focus on the highly abundant NMII, starting by discussing its biochemical properties, structural organization and regulation, and formation of bipolar filaments. We will then explore the involvement of NMII in axonal biology, from neuronal polarization to growth cone motility and neurite outgrowth, to its spatial arrangement and function, both in the axon initial segment (AIS) and in the axon shaft.

## 2. NMII: From Structure to Regulation and Function

### 2.1. NMII Isoforms and Structure

**Figure 1:**
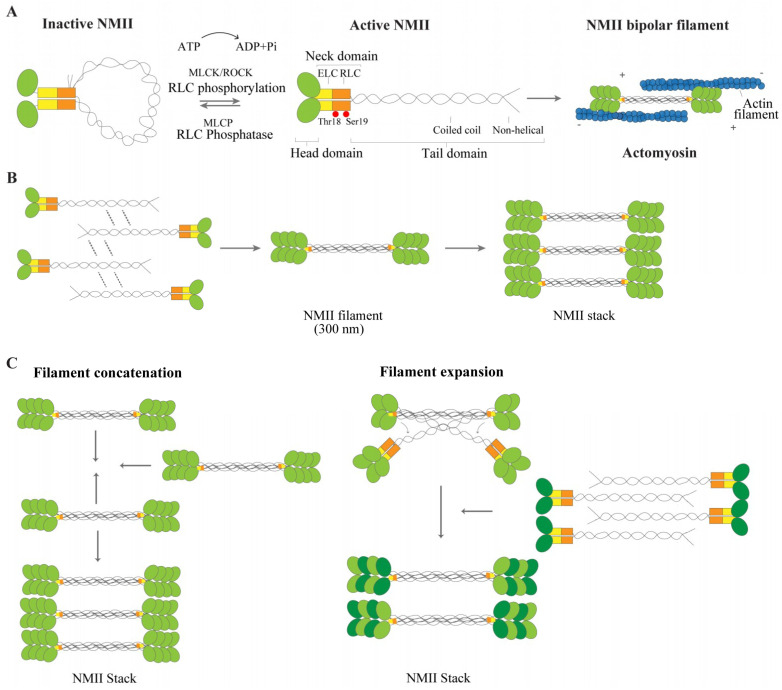
Non-muscle myosin II (NMII) structure and regulation. (**A**) NMII contains two heavy chains (HC), two regulatory light chains (RLCs) (orange) and two essential light chains (ELCs) (yellow). The heavy chain includes the head domain (green), with an actin binding site and an ATPase motor domain, the neck domain to which the ELC and RLC are bound, and the tail domain, with a helical coiled coil rod filament and a non-helical tail. In the absence of RLC phosphorylation, NMII is in an inactive conformation (left). Upon RLC phosphorylation by MLCK or ROCK, for instance, NMII unfolds to generate an active conformation (middle). MLCK phosphorylates RLC on Ser19 and Thr18, depicted as two red circles. The RLC phosphatase MLCP can revert this activation. NMII is then able to assemble into bipolar filaments, which bind to actin (blue) (right). (**B**) Upon NMII activation, polymerization-competent NMII molecules can form bipolar filaments through electrostatic interactions of their rod domains. Addition of more NMII molecules drives the growth of a bipolar NMII filament. For a matter of simplicity, a smaller number of myosins than that generally found on each side of the filament is drawn. (**C**) NMII filaments can also form super structures, the NMII stacks. Two models are considered to underlie NMII stack formation: Filament concatenation (left) and filament expansion (right). In the model of filament concatenation, NMII stacks can be formed through concatenation of multiple NMII filaments. In the model of NMII filament expansion, after the formation of the bipolar NMII filament, certain subset of daughter myosins separate themselves from one side of the filament, being each one used as a template. Growth of new templates is driven by addition of NMII filaments.

NMII is a hexameric protein composed by two regulatory light chains (RLCs) of 20 kDa and two essential light chains (ELCs) of 17 kDa, tightly bound to two heavy chains of 230 kDa (reviewed in [4,5]). Each heavy chain includes a N-terminal head domain (motor) and a long α-helical rod (tail domain), connected by a neck (lever arm) (Figure 1A) [4,5]. The head domains of NMII contain a binding site for ATP and also for actin. The neck region is important for the stability of the molecule. This region includes two conserved IQ motifs (IQxxxRGxxxR) that form an amphiphilic α-helix structure with binding affinity for the light chains (ELCs and RLCs). The neck domain is followed by a long α-helical coiled coil rod/tail filament, which mediates heavy chain dimerization and filament assembly. The molecule also includes a short non-helical tail of approximately 33–47 amino acid residues (Figure 1A), depending on the isoform [5]. Binding of light chains is required to maintain the structure of NMII and is critical for its proper function. As evidenced by X-ray analyses of different myosin crystals, the structure of the NMII lever arm is stabilized by the binding in tandem of ELC and RLC [6,7,8,9]. Supporting the importance of light chains for NMII stability, ablation or reduction of RLC expression levels triggers aggregation of NMII heavy chain in Drosophila [10].

In mammalian cells, NMII exists as three isoforms, NMIIA, NMIIB, and NMIIC [4,5,11,12,13,14,15,16] encoded by three different genes, *MYH9*, *MYH10,* and *MYH14*, respectively. The head domain is highly conserved between the different NMII isoforms, particularly in the actin-binding site. In contrast, both the C-terminal rod and the non-helical tail, that are crucial to determine the assembly of NMII filaments and intracellular location, differ significantly among the three isoforms [5,17]. The pattern of expression of NMII isoforms is cell type and tissue dependent, with few cell types expressing a single NMII. Generally, NMIIA and NMIIB are the predominant isoforms, while NMIIC is expressed in lower amounts. In neurons, expression of the three NMII isoforms has been reported, NMIIB being the predominant one [11,12,13,14,16,18]. Importantly, as will be further addressed below in the context of the axon, the activity, interactors and subcellular localization, may differ amongst the three NMII isoforms [19,20,21].

### 2.2. NMII Regulation and Function

NMII post-translational regulation has been widely covered in recent reviews [5,22,23] and is briefly discussed here. Phosphorylation of RLC is essential for the regulation of the enzymatic activity of NMII. The biological significance of this phosphorylation was initially unknown. In 1975, Adelstein and Conti showed that RLC phosphorylation by myosin light chain kinase (MLCK) isolated from platelets, increased the NMII ATPase activity [24]. Later on, it was demonstrated that this phosphorylation regulates the conformation of myosin heads, promoting the assembly of NMII filaments [25], the active unit in cells, as further detailed below. Beyond MLCK, several additional enzymes are able to phosphorylate the RLC on Ser19 and Thr18, such as Rho-associated coiled-coil-containing kinase (ROCK), leucine-zipper-interacting protein kinase (ZIPK), citron kinase, Serine/Threonine-protein kinase 21 (STK21) and myotonic dystrophy kinase-related CDC42-binding kinase (MRCK/CDC42BP) [25,26,27,28,29]. The above kinases display specific intracellular locations and are modulated by a variety of signal transduction pathways that provide an intricate regulation to accurately modulate NMII activity.

Addition of ATP to non-phosphorylated NMII favors the folded inactive monomer, in which the motor domains of the two heavy chains associate, and the helical tail folds back and interacts with the RLCs [30]. The folded monomer does not bind to actin therefore preventing the unnecessary consumption of ATP [5,31]. When phosphorylation of RLC occurs, the inactive conformation is disrupted allowing NMII to assemble into filaments, the active unit of the molecule (Figure 1A). To generate movement on actin, nanoscale motion of the NMII head domain is transmitted to the neck domain, which will ensure that the amplification of these movements is translated into a large power stroke [32]. NMII hydrolyzes one molecule of ATP into ADP and P_i_, and the neck domain changes from a bent to a straight conformation [32]. Consequently, the neck domain rotates generating a power stroke. As a result, an actin filament translocates 5 to 10 nm [33].

RLC phosphorylation is a reversible biochemical process, tightly regulated by several myosin kinases and phosphatases. In the latter case, a RLC phosphatase will decrease NMII activity by favoring the inactive conformation [34]. RLC phosphorylation at Ser19 and Thr18 is reverted by myosin light chain phosphatase (MLCP) (Figure 1A), which is a multi-protein complex including a catalytic subunit and a myosin-binding subunit encoded by *PPP1R12A* and referred to as myosin phosphatase target subunit 1 (MYPT1) [35,36]. MYPT1 phosphorylation inactivates protein phosphatase 1 (PP1) and this leads to a significant increase in RLC phosphorylation and NMII activation [34].

Two important kinetic properties that differ among NMII isoforms are the ATPase activity, and the duty ratio, i.e., the time that the myosin motor domain remains strongly bound to actin [37]. NMIIA presents the highest rate of ATP hydrolysis and it pushes forward actin filaments more rapidly than NMIIB and NMIIC [38]. In contrast, NMIIB has a significantly higher duty ratio than NMIIA and NMIIC, spending more time bound to actin [39]. These observations support the concept that NMIIA and NMIIC may be more involved in contraction of actin filaments, while NMIIB can also maintain tension by crosslinking actin filaments [39]. A *MYH10* mutation (R709C), that is located in the head domain, allows the separation of these two functions, providing insight into how NMIIB acts. Although this mutation exhibits very slow ADP release and a disruption in the ability to translocate actin, it spends most of time in a strong-binding state to actin filaments during the ATPase cycle [38,40].

### 2.3. NMII: From Filament Formation to Stacks

The C-terminal tail domains of NMII heavy chains interact electrostatically with each other to form bipolar filaments, positioning the N-terminal head domains on opposite sides of the filament (Figure 1B). Early studies using electron microscopy revealed that the length of the NMII bipolar filament is approximately 300 nm [41,42], just above the diffraction limit of standard light microscopy. This length was later confirmed using 3D-structured illumination microscopy [43]. Limitations in imaging techniques underlie the fact that questions related to the number of NMII molecules necessary to assemble NMII filaments, and the process of assembly itself, remain unresolved. A single filament includes multiple NMII molecules and the number of molecules is dependent on steric hindrance [41,44]. NMIIA and NMIIB assemble into filaments of approximately 30 monomers, while NMIIC assembles into filaments of fewer molecules, approximately 14 monomers [41,42]. This corroborates Thomas Pollard’s initial findings on in vitro recombinant NMII isoforms using electron microscopy [42]. Most NMII filaments are thought to be homofilaments. However, in areas where NMII isoforms (NMIIA, NMIIB and NMIIC), are simultaneously expressed, the assembly of heterofilaments has also been observed [45,46]. Bipolar filaments can additionally form superstructures made of groups of parallel filaments termed as NMII ribbons or stacks [47,48,49] (Figure 1B). The mechanisms governing their formation are still unclear. NMII filaments may associate amongst each other, in a process known as concatenation [43,44,47] or alternatively they may undergo splitting, with a single filament giving rise to two separate daughter filaments, in a process known as expansion (Figure 1C) [44].

## 3. Actomyosin in Neurons

Neurons are one of the most highly polarized cells in our bodies and their exquisite shape is crucial for the physiology of the nervous system. Neurons possess distinct compartments, the dendrites and the axon that extend from the cell body (soma). Whereas dendrites receive signals from other neurons, the axon transmits signals through the release of neurotransmitters. Typically, the axon is a single long and thin process, also entailing different compartments, with specific functions and structural organizations. These include the axon initial segment (AIS) located close to the cell body, the place where action potentials are generated, the axon shaft, and in the distal axonal tip, the growth cone (during development) or the pre-synaptic terminal (following the establishment of connections). In this review we will focus on the role of NMII in the biology of the different axonal compartments, starting from its outermost region—the growth cone—up to the AIS.

### 3.1. NMII: From Neurite Formation during Neuronal Polarization to Axon Regeneration in the Adult

#### 3.1.1. The Actomyosin Cytoskeleton in the Growth Cone

How a round cell breaks symmetry and gives rise to a highly polarized neuron has fascinated neuroscientists for decades. At the tip of the axon and dendrites, growth cones are able to sense and integrate a variety of signals inducing changes in cytoskeletal dynamics, which will ultimately guide them to their targets. The morphological changes that occur during neuronal development in vivo can be recapitulated to some extent in vitro at least for certain neuron types [50,51]. The importance of the actin and microtubule cytoskeletons in neuronal polarization and growth cone formation have been extensively investigated (reviewed in [52]), as well as how intrinsic and extrinsic cues modulate these processes. Here we will focus our attention on the role NMII in the growth cone.

**Figure 2:**
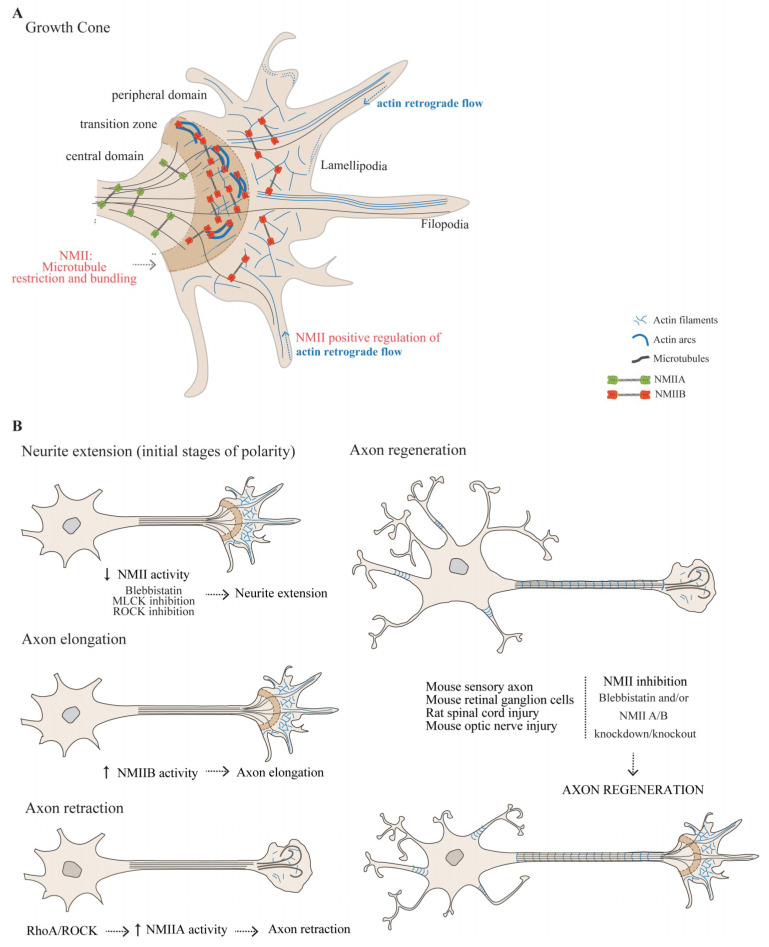
NMII in axon elongation, retraction and regeneration. (**A**) Schematic representation of the growth cone. The growth cone includes a central domain, mainly consisting of bundled microtubules (grey lines), a transition zone, enriched in actin arcs (blue hemicircular lines) and a peripheral domain, enriched in actin (blue lines) in the form of lamellipodia and filopodia. Actin bundles assemble near the growth cone leading edge, translocate rearward by retrograde flow towards the transition zone (blue dashed arrows in the peripheral domain) and recycle through bundle severing. NMIIA (green) is mainly located within the central domain and NMIIB (red) in the transition zone and peripheral domain. (**B**) General effect of NMII in neurite extension, axon elongation, retraction and regeneration. During early neurite extension pharmacological inhibition of NMII either directly using blebbistatin, or indirectly through the inhibition of the upstream regulators MLCK or ROCK, promotes axon elongation (upper left panel). When analyzing the effect of different NMII isoforms in axon elongation, in general terms, NMIIB is required for axon elongation (middle left panel) while NMIIA participates in neurite retraction (bottom left panel). In the course of axon regeneration (right panel) NMII inactivation through blebbistatin and/or NMIIA/B downregulation/knockdown, enhances axon regeneration.

The growth cone is a highly dynamic structure, comprising a central domain, a transition zone and a peripheral domain [53] (Figure 2A). The central domain consists mostly of stable, bundled microtubules whereas the peripheral domain is enriched in actin, either in the form of fingerlike filopodia that dynamically explore the environment for guidance information, or lamellipodia whose turnover contributes to the forward movement of the growth cone. The transition zone is located at the interface between the peripheral and central domains, and is enriched in actin arcs, forming a hemicircumferential ring (Figure 2A). The transition zone may restrain dynamic microtubules from protruding into the peripheral domain [54,55]. The continuous rearward movement of F-actin from the leading edge towards the growth cone center—actin retrograde flow, combined with F-actin treadmilling i.e., the addition of actin subunits to the barbed-end and disassembly from the pointed-end, is essential for growth cone response to directional cues [53].

The role of NMII in growth cone organization and dynamics, namely in actin arc formation and movement, peripheral domain actin retrograde flow, actin bundle severing in the transition zone, and axon guidance, has been extensively investigated [56,57,58,59,60,61]. Although NMII is enriched in actin arcs in the transition zone, the development of antibodies against specific NMII isoforms allowed to determine that the three NMII isoforms are differentially distributed throughout the growth cone [58]. While NMIIA is highly expressed in the axon shaft and central domain [58], NMIIB and NMIIC are enriched in the transition zone and in the peripheral domain [58,59,60]. Using electron microscopy, both NMIIA and NMIIB, were found in the growth cone in their active form i.e., as bipolar filaments [56]. As is detailed below, the distinct spatial organization of NMII isoforms within the growth cone may underlie the apparent opposite roles of NMIIA and NMIIB in growth cone actin retrograde flow, guidance and axon growth.

In the transition zone, actin arcs function as NMII-driven contractile structures. Using structured illumination microscopy, NMII filaments in actin arcs were shown to be oriented parallel to actin filaments and to co-localize with regions enriched in actin pointed ends, associated with tropomodulin [48]. Actin arc movement is decreased by ROCK and MLCK inhibition and increased after MLCP inhibition, which is consistent with actomyosin-dependent contraction [61]. Actin arc formation is also sensitive to changes in Rho activity [53,61], and is compromised by NMII inhibition through blebbistatin [53,57]. In fact, when NMII activity is inhibited by blebbistatin, long parallel actin bundles fill the complete growth cone, leading to expansion of the peripheral domain, while NMII disappears from the transition zone [53,57]. Of note, actin arcs interact with microtubules and transport them back into the central domain, as NMII provides for the compressive force necessary for microtubule bundling in the growth cone neck [62].

In the peripheral domain of the growth cone, actin retrograde flow is central for axon guidance. Actin bundles assemble near the growth cone leading edge, translocate rearward by retrograde flow, and recycle through bundle severing. Although the role of NMII in the retrograde flow was initially questioned [63,64], several evidence support that this process is indeed NMII-dependent. Actin retrograde flow is significantly decreased by ATPase inhibitors including 2,3-butanedione-2-monoxime (BDM) [65] and blebbistatin [57], as well as by MLCK inhibitors [61]. The steady-state “treadmilling” of actin filaments maintains actin bundles at relatively constant lengths. When NMII activity is inhibited by blebbistatin, the actin bundle length increases significantly and the severing process within the transition zone is impaired [57]. These observations suggest that actin retrograde flow rate in the growth cone is positively regulated by the activity of NMIIB, the isoform more abundant in the peripheral domain and in the transition zone. However, when actin retrograde flow of NMIIB-knockout mouse growth cones was investigated, an increased rate was found, which was suggested to be due to a functional takeover by other NMII isoforms [66].

#### 3.1.2. The Role of NMII in Growth Cone-Mediated Axon Elongation

Inhibition of NMII activity in primary neuron cultures and in neuronal cell lines has disclosed its importance in axon elongation and retraction. During the initial stages of neuronal polarity, extension of undifferentiated minor processes occurs. These will then differentiate into the axon and dendrites which will elongate in a growth cone-dependent manner. Inhibition of NMII with either blebbistatin, or through the inhibition of its upstream regulators (MLCK or ROCK), promotes the fast growth of minor processes [67] (Figure 2B). These data indicate that NMII negatively regulates neurite outgrowth in the early stages of neuronal polarization. When actin polymerization is inhibited by latrunculin, neurite extension is further potentiated with concurrent NMII inhibition, but fails to reach the magnitude of extension produced by blebbistatin alone [67]. These data suggest that NMII negatively regulates neuronal development by generating contractile forces against F-actin. When microtubule polymerization is disrupted through the use of nocodazole, the increase in minor process length induced by blebbistatin is prevented, indicating that microtubule dynamics is required for blebbistatin-induced neurite outgrowth [67].

Based on classical experiments using 2D neuronal cultures, following the establishment of the growth cone, axon elongation occurs through a molecular “clutch” that links the substrate to the actin cytoskeleton in the growth cone [68]. This attachment to the substrate is thought to be needed for the axon to exert forces necessary for axon extension. The molecular “clutch” is a three-step process sequentially involving *protrusion* and substrate-attachment of filopodia and lamellipodia, followed by the *engorgement* of the growth cone by microtubules and organelles, and finalized by the suppression of protrusive activity, and *consolidation* of a new stretch of stable axon shaft behind the advancing growth cone. In this model, axon growth is mediated by the growth cone that pulls itself and the axon along the substrate through actomyosin-mediated contraction [69,70]. NMII-based contractility restricts microtubules from engorging the growth cone [53,65,71,72,73]. Accordingly, when NMII activity is inhibited by blebbistatin, filopodia in the peripheral domain present an increased number of microtubules [74]. Interestingly, microtubules can penetrate from the central domain towards the peripheral domain of the growth cone only if the forces generated by the retrograde motor dynein, overcome the NMIIB-driven forces [75].

When axon growth takes place in the presence of the permissive substrate laminin, inhibition of all NMII activity decreases axon elongation (reviewed in [71]). When analyzing the effect of different NMII isoforms in axon extension, several evidence support that in general terms, NMIIA participates in neurite retraction, while NMIIB is required for neurite outgrowth (Figure 2B). In line with this view, when antisense oligonucleotides against NMIIB are used in neuroblastoma cells, a significant decrease in outgrowth takes place [60]. Moreover, NMIIB knockout superior cervical ganglia neurons have decreased rates of axon outgrowth [76] and axon growth is impaired in *MYH*10 knockout mice [71]. In relation to NMIIA, an important body of data supports the role of the RhoA/ROCK pathway as an upstream activator of this NMII isoform, modulating its ability to repress axon growth [61,77,78,79,80]. Inhibition of NMIIA activity results in actin rearrangement in the growth cone and in the loss of focal contacts that culminates in increased axon growth [79,81,82,83]. Additionally, it has been recently suggested that RhoA may inhibit axon growth by activating NMII in the actin arc, preventing microtubule protrusion towards the leading edge of the growth cone [54], which is essential for axon growth. Together these data suggest that a tight regulation of NMII activity in the transition zone is necessary to enable a balanced microtubule entry into the peripheral domain, compatible with optimal axon growth.

In summary, NMII regulates several aspects of axon extension, ranging from actin retrograde flow, growth cone engorgement by microtubules, and substrate adhesion. How these different functions are integrated and tuned by the different NMII isoforms during the process of axon growth, remains to be fully clarified. Despite the dominant view that neurons need adhesions to extend axons through actomyosin-mediated pulling force, one should however bear in mind that the molecular clutch model should probably be revisited. Very recently, it has been demonstrated that in the more physiological environment of 3D matrices, growth cones can extend axons independently of adhesions and pulling forces on their substrates [84]. In 3D, microtubules were shown to grow unrepressed by the actomyosin cytoskeleton into the growth cone peripheral domain, enabling a fast amoeboid-like axon elongation. Thus, whereas axon growth in 2D is enhanced by actin destabilization or inhibition of the actomyosin cytoskeleton, this is apparently not the case for axon growth in 3D. This novel view on axon elongation should certainly be further explored in the future.

#### 3.1.3. NMIIA and NMIIB Play Central Roles in Axon Guidance

Neurons are able to respond to both attractant and repellent guidance cues that are translated into alterations in cytoskeletal dynamics leading to changes in growth cone motility, direction and growth rate. Many axon-guidance cues affect the contraction of NMII by modulating the balance between Rho, Rac, and Cdc42 activities [57,85]. The combination of individual NMII isoforms and guidance cues is important for each neuron to grow in a given direction. Although NMII isoforms are quite similar in structure and are capable of partially replacing each other [86], each NMII isoform has been implicated as playing different roles in axon guidance, in a context-dependent manner. As explored above, while NMIIA is generally thought to be responsible for neurite retraction, NMIIB is required for neurite outgrowth as a response to positive guidance cues [60,71,76,79]. Accordingly, NMIIA knockdown promotes neurite outgrowth while NMIIB knockdown inhibits outgrowth on permissive cues such as poly-L-lysine and laminin [59]. Work by Turney and colleagues has nicely shown how the substrate dictates the organization of NMII isoforms within the growth cone, modulating the balance of tension forces [87]. The authors have demonstrated that NMII activity is required for faster axon elongation in response to nerve growth factor (NGF). This occurs through the regulation of two actomyosin-dependent mechanisms: Transverse actin bundling and actin retrograde flow that oppose microtubule advance. In the presence of the permissive substrates laminin and fibronectin, NMIIA and NMIIB display differential roles [87]. Whereas large stable adhesions on fibronectin enhance NMIIA-dependent transverse actin bundling, small transient adhesions on laminin promote NMIIB-dependent slowdown of actin retrograde flow [87]. This is in accordance with the fact that NMIIB KO cells present weaker traction forces on laminin and a more undirected growth cone advance [88], as well as a higher rate of actin retrograde flow [71]. In the absence of NMII activity, NGF failed to stimulate axon elongation, supporting its importance in axon outgrowth [87].

The role of NMIIA and NMIIB in the response to inhibitory molecules may be more complex than that observed with attractive cues. When NMIIB knockout neurites are grown in alternating patterns of permissive and non-permissive guidance cues (laminin-1 and poly-L-ornithine, respectively), the absence of NMIIB enable neurites to cross the barriers without changing direction [59]. In the case of inhibitory chondroitin sulfate proteoglycans (CSPGs), knockdown of either NMIIA or NMIIB reduces axon growth capacity [81,89,90]. In response to the non-permissive cue semaphorin 3A, NMIIA and NMIIB are involved in growth cone collapse and neurite retraction, respectively [91]. NMIIA also mediates growth cone collapse and neurite retraction in response to repulsive guidance molecule (RGMa) [82]. In the case of ephrin-A5, a non-permissive molecule, by binding to EphA3 it triggers the activation of RhoA/ROCK activating NMIIA and ultimately leading to axon repulsion [92]. This context-specific role of NMIIA/B response to different guidance cues is certainly the subject of the intricate regulation, balance and spatial distribution of both NMII isoforms.

#### 3.1.4. NMII as a Modulator of Axon Regeneration in the Adult

Following the establishment of synapses, mammalian central nervous system (CNS) axons fail to recapitulate development and are generally unable to regrow after injury. In the last decades, many mechanisms underlying axon regenerative failure have been identified. From the neuronal intrinsic standpoint, following lesion, axons need to induce local cytoskeleton remodeling to promote the formation of a new growth cone [93,94]. Several reports support a strong link between NMII inhibition and increased axon regeneration after injury. In rats, after spinal cord injury, phosphorylated MLC is up-regulated in axons close to the lesion site, in a Rho-dependent manner, and growth cone collapse is mediated by NMIIA [82]. Supporting the causative role of NMIIA in hampering axon regrowth, silencing its gene promotes axon regeneration after contusive spinal cord injury in rats [95]. In regenerating mouse sensory axons, when NMIIA and NMIIB are knocked down or pharmacologically inhibited by blebbistatin, axon regeneration occurs irrespectively of the presence of inhibitory cues including CSPGs and myelin-based inhibitors [81]. Inhibition of NMIIA and NMIIB results in loss of lamellipodia and actin arcs, causing significant microtubule protrusion towards the leading edge of the growth cone. As a result, axon growth rate over non-permissive substrate is accelerated [81]. Likewise, in vivo studies using double knockout mice for NMIIA/NMIIB in retinal ganglion cells showed increased optic nerve regeneration [96]. When the growth cone morphology and axon trajectory were analyzed, the absence of NMIIA and NMIIB abolished almost completely the formation of retraction bulbs, thus enhancing axon extension efficiency [96]. In contrast, in goldfish retinal ganglion cells, MLCK, the kinase that triggers RLC phosphorylation leading to the formation of active NMII bipolar filaments, is upregulated in regenerating axons [89]. In this system, if NMII activity is inhibited through the use of MLCK inhibitors (ML7 or ML9), growth cones of regenerating axons cease to move. This result indicates that, in contrast to mice, NMII activity is needed in goldfish retinal ganglion cells for successful axon regeneration [89]. It is therefore possible that in different species, the modulation of NMII activity generates different outcomes in axon regeneration.

### 3.2. Distribution of NMII Throughout the Axon Shaft: From Enrichment in the AIS to Its Presence Throughout the Axon Shaft

Beyond its function in the growth cone, the existence of active NMII in the AIS [97,98] and throughout the axon shaft [99,100] has recently gained attention. Here we will discuss the potential functional consequences of NMII enrichment in the AIS as well as its role and possible structural organization in the axon shaft.

#### 3.2.1. Why Is Active NMII Enriched in the AIS?

The AIS is a highly organized region generally located in the proximal axon of neurons. It has a specialized cytoskeletal architecture, central for the generation of action potentials and for the establishment of neuronal polarity. The AIS displays structural plasticity, as it can change its length and location relative to the soma, in an activity-dependent manner, fine-tuning neuronal excitability [101]. Using rat hippocampal neurons, the downstream mechanisms enabling structural changes at the AIS begun to be unveiled, as both its long-term relocation and rapid shortening could be blocked by blebbistatin [98]. This initial data established a link between NMII and AIS function, suggesting that its primary role at the AIS might be to enable activity-dependent morphological alterations [98].

**Figure 3:**
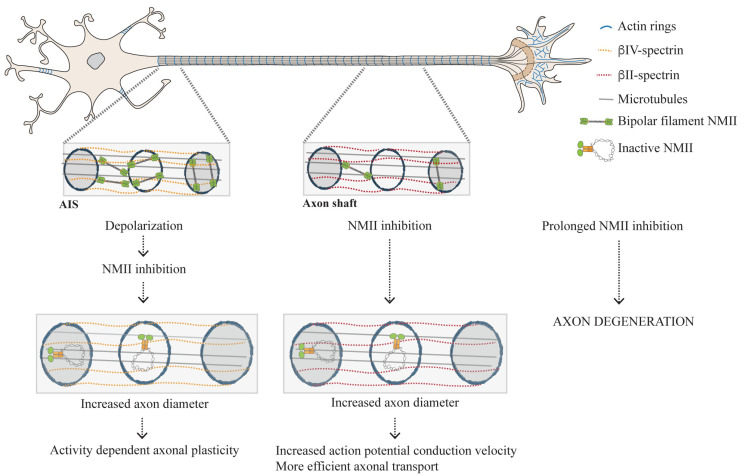
Schematic representation of the role of the actomyosin cytoskeleton in the control of axonal contractility. Phosphorylated myosin light chain is highly enriched at the AIS, where it may participate in the regulation of axon diameter during action potential firing. In the axon shaft, circumferential and longitudinal axon tension are also regulated by the actomyosin network. While pMLC forms circular periodic structures colocalizing with membrane periodic skeleton (MPS) actin rings, NMII heavy chains appear mostly distributed as multiple filaments with approximately 300 nm of length along the longitudinal axonal axis, crosslinking adjacent actin rings (probably providing for tension); NMII filaments spanning individual rings may also exist (and provide for contraction).

Later, it was demonstrated that pMLC and NMII activity are necessary and sufficient to initiate AIS assembly [97]. Although pMLC is initially abundant and uniformly distributed throughout the axon (DIV2), it accumulates very early at the AIS simultaneously with ankyrin G (the prototypic AIS marker normally considered the prime nucleator of its assembly) [97] (Figure 3). An asymmetric distribution of NMII kinases and phosphatases was suggested to underlie pMLC enrichment at the AIS [97]. From evidences collected using STORM nanoscopy, it was proposed that pMLC associates with actin rings [97] within the axonal membrane periodic skeleton (MPS) [102]. The MPS, a highly regular network composed of actin rings spaced by spectrin tetramers approximately every 190 nm, is thought to maintain axonal structural integrity [103,104]. More recently, tropomyosin 3.1 (Tpm3.1) was also shown to be necessary for the structural and functional maintenance of the AIS probably by recruiting NMIIB to this structure, thus mediating its possible contractility [105].

Interestingly, pMLC is rapidly lost from the AIS during neuronal depolarization, via Ca^2+^-dependent mechanisms, leading to destabilization of the actin cytoskeleton [97]. This finding furthers elucidates the mechanism of activity-dependent structural plasticity of the AIS (Figure 3). Of note, it has long been know that axon diameter is regulated by activity-dependent mechanisms as axons swell during the generation of an action potential (reviewed in [106] and [107]). Later, NMII activity was also shown to be involved in axonal electrophysiology as blebbistatin increases action potential conduction velocities in hipppocampal neuron cultures [99]. This supports that in addition to modulating activity-dependent structural plasticity of the AIS, NMII may also be involved in the regulation of axonal conduction. One should bear in mind that the role played by NMII in the AIS, that is probably related to its contractile properties and ability to rearrange the actin cytoskeleton, as well as its spatial distribution in this axonal compartment are just starting to be unveiled.

#### 3.2.2. The Actomyosin Cytoskeleton as a Key Regulator of Circumferential and Longitudinal Axonal Tension

Although pMLC is enriched in the AIS [97], it is also found throughout the axon shaft [99,100] (Figure 3). The possible interplay between actin rings in the axon shaft, as potential anchors for NMII filaments, was explored by independent groups that reached similar complementary results [99,100,108]. Through the use of chemical inhibitors (blebbistatin [99,100,108], ML7 [99,100,108], and Y-27632 [108]) as well as shRNA-mediated downregulation, decreased NMII activity/expression was shown to lead to increased axonal diameter [99,100,108]. Whereas downregulation of NMIIA and NMIIB produced similar effects, NMIIC knockdown did not affect axonal caliber, suggesting that this specific NMII isoform is not involved in the regulation of axonal diameter [99]. Together, the data gathered by the above-referred groups support that the actomyosin cytoskeleton participates in the generation of circumferential tension along the entire length of the axon shaft. Conceptually, this finding has important implications in axonal biology as by regulating radial contractility, NMII impacts action potential conduction [99], the efficiency of axonal transport [100] and possibly on the onset of axon degeneration as a sustained NMII inactivation disrupts the MPS entailing the formation of focal axonal swellings [100] (Figure 3).

Using super resolution microscopy, pMLC was shown to be organized in a circular, periodic conformation colocalizing with MPS actin rings, intercalating with βII-spectrin [99]. In turn, NMII heavy chains appeared distributed as multiple filaments with approximately 300 nm of length along the longitudinal axonal axis [99]. NMIIA heavy chains showed sites of colocalization with βII-spectrin [99], whereas NMII head domains colocalized more extensively with periodic actin rings [99,100]. This supports that NMII filaments can crosslink adjacent rings, as previously suggested by platinum-replica electron microscopy [109] (Figure 3). Occasionally, NMII heavy chains colocalized with phalloidin, indicating that these may also be located within individual actin rings [99,100] (Figure 3). NMII filaments within single actin rings are in principle the conformation that is able to generate the highest contractile force. In contrast, NMII filaments crosslinking adjacent actin rings are not expected to provide for radial contractility but provide for scaffolding. The details of NMII filament composition and structural organization in the axon shaft are just starting to emerge. The nature of their interaction with actin (with MPS actin rings or even with deeper axonal actin structures), together with a more profound understanding of their spatial distribution, will certainly bring new light to our knowledge on the fluctuations in axonal diameter occurring during axonal transport, action potential firing and conduction, and axon degeneration. One should also take into account that recently, the MPS actin rings were suggested to be made of two long parallel intertwined actin filaments [109]. This unusual actin arrangement poses exciting questions on how such filaments might be able to dynamically adapt to oscillations in axonal diameter, and how NMII activity could orchestrate for their contraction.

In addition to controlling axon diameter, NMII is also central in modulating longitudinal axonal tension. In drosophila, NMII knock down or NMII reduced activity through treatment with ML7 or Y27632, lead to reduction in longitudinal axonal contraction [110]. In chick DRG neurons, the NMII inhibitor blebbistatin also induces a similar effect as it blocks axon straightening upon trypsin-induced de-adhesion [111]. Of note, when considering the longitudinal organization of the axonal cytoskeleton, neither drug- nor shRNA-mediated modulation of NMII activity, result in alterations of MPS periodicity [99,100,111]. Our knowledge on NMII positioning along the axon will certainly evolve in a manner that will allow us to understand its participation in axonal radial contractility and longitudinal tension, and comprehend how NMII activity does not interfere with the length of the extended MPS spectrin tetramers.

## 4. Conclusions

The actomyosin cytoskeleton contributes to various essential cellular processes. Although the NMII structure, regulation and function in diverse cell types have received intense attention, there are still several open questions related to the organization and role of actomyosin networks in neurons. What is the interplay between different NMII isoforms that allows them to perform independent functions during axon growth and growth cone guidance? How can we reconcile these different functions with the fact that under specific circumstances they may replace each other? Is it possible that NMII regulates axon growth not only in a growth cone-mediated manner but also by involving the actomyosin networks present in the axon shaft? What is the spatial distribution and structure of NMII in axons and how does it accommodate for the simultaneous regulation of axon longitudinal and radial tension? There are also unanswered questions as to the involvement of NMII in brain dysfunction. As detailed above, NMII is a crucial player during different stages of neural development. Accordingly, NMII mutations have been associated with several neurodevelopmental disorders as reviewed before [112]. Beyond development, NMII may also contribute to neurodegenerative diseases such as Alzheimer’s disease [113,114,115,116]. In this disorder, among other mechanisms, NMIIB can exert a tensional pressure on the F-actin network that may impair amyloid precursor protein translocation towards the cell membrane [113]. In the context of neurodegenerative disorders, disassembly of the axonal MPS (an actomyosin network), has been implicated in trophic deprivation-mediated axon degeneration [117,118]. Further reinforcing the involvement of NMII in degeneration, prolonged NMII inactivation leads to disruption of periodic MPS actin rings and to the formation of focal axonal swellings [100]. In this respect, could NMII be used as a therapeutic target to revert axonal swellings and axon degeneration? With the recent and powerful advances on super-resolution microscopy and correlative techniques, these and other questions will certainly be an exciting area to explore in the coming years.
